# Billboard advertising: an avenue for communicating healthcare information and opportunities to disadvantaged populations

**DOI:** 10.1186/s12913-017-2754-6

**Published:** 2017-12-13

**Authors:** James K. Elrod, John L. Fortenberry

**Affiliations:** 1 Willis-Knighton Health System, 2600 Greenwood Road, Shreveport, LA 71103 USA; 2 LSU Shreveport, 1 University Place, Shreveport, LA 71115 USA

**Keywords:** Billboard advertising, Health communication, Community health, Medically underserved populations

## Abstract

**Background:**

Healthcare communications directed toward the disadvantaged have the potential to elevate the health status of these underprivileged and highly-challenged individuals. From conveying advice which encourages healthy lifestyles to communicating the location and availability of various medical resources, healthier lives and communities can be realized. Success on this front first requires establishing an effective communications link, something that is made more difficult as communications options available to the disadvantaged are more limited than those available to advantaged populations.

**Discussion:**

One avenue which shows exceptional promise for successfully engaging the disadvantaged is that of billboard advertising. Willis-Knighton Health System’s experiences and insights indicate that the characteristics and qualities of billboards, paired with the environmental circumstances typically faced by the less fortunate, create unique combinations which amplify consumption of billboard advertising content. Further, research suggests that the less privileged place greater reliance on the medium than do their more privileged counterparts, escalating the value and impact potential of billboard advertising directed toward the disadvantaged.

**Conclusions:**

Given the value afforded by health and wellness information successfully reaching the disadvantaged, opportunities to better distribute content to targeted audiences could very well improve community health. Billboard advertising appears to be well suited to engage the less fortunate, providing a productive pathway for the conveyance of helpful, supportive details, yielding healthier populations, enhanced opportunities, and better communities.

## Background

Healthcare communications directed toward the disadvantaged play a critical role in the lives of these underprivileged and highly-challenged individuals [1–4]. By successfully conveying the availability of a medical service at a hospital, the benefits of embracing a healthy lifestyles practice, the need to visit a medical clinic for health screening, and similar things of benefit and value to the underserved, healthcare entities can encourage actions which elevate health and wellness outcomes and the resulting quality of life achieved by the less fortunate. As communications typically serve as prerequisites for awareness, attention, and action on the part of sought audiences, excellence on this front is a must for healthcare organizations seeking to engage and inform the poor [2, 5].

Successful healthcare communications, of course, require reaching these disadvantaged groups effectively, permitting conveyance of information which ideally will be understood, acknowledged, and embraced, affording opportunities for the realization of healthier lives and communities. In engaging underserved populations, however, difficulties abound as their communications options are more limited than those available to more advantaged individuals [1, 6]. In addition to being medically underserved, impoverished populations typically suffer from reduced social status, low or even no income, inadequate or nonexistent housing, and reduced education levels, among many other hardships that diminish opportunities for interaction and engagement [7–12]. While this particular challenge—limited means of communication—often takes a back seat to more pressing issues faced by the disadvantaged, it is no less important as reduced or nonexistent exposure to messages concerning community health resources, healthy lifestyle practices, and related items can result in missed opportunities which, in turn, can lead to a diminished health status [1–4, 6].

Limited communications options on the part of the indigent indeed serve as significant barriers which hamper charitably-minded healthcare providers as they pursue their given missions. Such obstacles, however, should not be discouraging. Instead, providers must rise to the challenge and think creatively in a bid to identify prudent pathways which reach targeted groups. As reliance on traditional methods of communication to connect with the impoverished may or may not be adequate, those desirous of successfully reaching these challenged groups must be open to investigating and pursuing prudent avenues for doing so, whatever they may be. Indeed, addressing such groups proficiently requires obtaining knowledge and insights on how best to connect with them [1, 2, 5].

Shreveport, Louisiana-based Willis-Knighton Health System, through its extensive experience deploying a range of advertising media, supplemented by insights from the literature regarding the latest marketing communications innovations, has observed billboard advertising to be a particularly productive avenue for connecting with disadvantaged audiences. Willis-Knighton Health System’s use of billboards is extensive and historic. Known for adopting innovations emerging outside of the healthcare industry for deployment within [13], in the 1970s, Willis-Knighton Health System became the first hospital in Louisiana to make use of billboards to market healthcare services [14]. At present, the system leases 28 billboards in the greater Shreveport marketplace, more than any other healthcare provider in the region. As market leader, Willis-Knighton Health System deploys billboards strategically in a variety of ways as a means of engaging audiences ranging from mass markets to distinct niches. It has acquired practical insights for communicating effectively with its customer groups, including those of limited means.

The characteristics and qualities of billboards, paired with the environmental circumstances typically faced by the less fortunate, create unique combinations which amplify consumption of billboard advertising content. Further, research indicates that the less privileged place greater reliance on the medium than do their more privileged counterparts, escalating the value and impact potential of billboard advertising directed toward the disadvantaged [15, 16]. While these insights are beneficial for most any healthcare establishment which offers at least some degree of charity care, they hold especially high value for those entities which focus exclusively on addressing disadvantaged populations. As billboard advertising as a means of connecting healthcare-related content with underserved populations perhaps would not ordinarily come to mind, insights regarding the medium, its characteristics, and impact on the impoverished can be most helpful for advancing health communications directed toward this difficult to reach audience.

## Overview and attributes

Billboard advertising is a particular type of communication involving the use of large, stationary structures placed along roadsides and other transit routes to display messages to passersby [17]. It is a component of the advertising category known as outdoor or out-of-home advertising which seeks to engage audiences as they circulate about communities [18]. As with most any form of advertising, billboard advertising is used to communicate with current and prospective customers, with the usual goal being to entice them into some sort of exchange [17–19]. The term *billboard* comes from the medium’s early origins where handbills (i.e., papers containing promotions of some sort) were posted onto boards (i.e., wooden panels erected for the purpose of displaying handbills) to inform passersby of events, product promotions, and other advisories [20, 21]. While the medium no longer involves posting handbills onto boards, its original descriptor remains. Today, the content displayed on billboards often is digitally printed onto vinyl and applied manually to given structures, but increasingly billboards are being retrofitted with electronic display panels which permit vibrant images to be rotated on demand via a computer interface. This also allows single billboard locations to support multiple messages, rotated in succession, affording advertisers with opportunities to present a range of content at given locations, increasing the communications potential of the medium [18, 22].

Billboard advertising is positioned nicely to capitalize on a number of changes associated with societal developments and advancements. Notably, the number of miles traveled via roadway by consumers continues to increase [23], affording greater opportunities for exposure to billboard advertisements. Additionally, ad-blocking technologies, which permit audiences to erase advertisements from view, do not exist with billboard advertising, further increasing exposure opportunities. The medium also carries significant value, as billboard advertising offers the lowest CPM (i.e., cost per thousand, a measure of the cost necessary for an advertisement to reach 1000 individuals) of the major media categories [15]. This noted collection of attributes should see billboard advertising become an increasingly important medium of communication over time [15, 18, 22].

## Segmentation potential and impact on the disadvantaged

Although often viewed merely as a tool for reaching broad audiences with little regard for precision, billboards actually can be used somewhat surgically to connect with desired audiences. Its segmentation potential—the ability of a medium to be directed effectively to engage a group of individuals sharing common characteristics—actually is quite good and, in some cases, can be excellent, depending on the particular audiences sought [16]. The disadvantaged, in fact, happen to be a population which billboards can target exceptionally well. This excellent segmentation potential emerges because of two primary reasons:

- Contextual matters which place billboard advertisements and many disadvantaged populations in close proximity to each other, and
- Inherent receptiveness on the part of underserved populations for billboard advertisements and the informational value they provide.

Contextually, outdoor advertising firms seek to place billboards at locations with the highest traffic counts in given marketplaces as a means of increasing the exposure potential of the featured advertisements [18, 21, 22]. This makes high-density, urban areas top choices for billboard placements. While most of the audiences encountering billboards have only fleeting exposure (e.g., transiting by them as they pursue their ultimate destinations), those residing in close proximity to given displays face constant exposure to the associated advertising messages. In scores of municipalities across America, inner-city neighborhoods tend to be most heavily occupied by those of limited means, placing them at the nexus of billboard density. This naturally results in exposure levels to billboards which far exceed those of their more privileged counterparts who reside in less dense and often more affluent locations, many of which prohibit billboards for reasons of aesthetics or other factors [24–26]. Further, with limited or nonexistent financial resources, poverty-stricken individuals have fewer sources of information at their disposal, giving the free and readily available content supplied by billboards an increased opportunity to engage and influence. Additionally, as the less fortunate more heavily rely on public transportation, exposure to billboards is magnified as city buses typically follow the most highly traveled routes in communities, with these often being heavily laden with billboards [16]. All told, when reflecting on the environments which the disadvantaged typically call home, opportunities for exposure to billboards are epic and difficult to ignore.

Historically, the intensive exposure of underserved populations to billboards has been viewed to be troubling by many. High exposures of the disadvantaged to billboard advertisements featuring and often glamorizing unhealthy products, such as alcoholic beverages, foods and drinks associated with obesity, and the like certainly are cause for concern [27–30]. In fact, associated concerns were so pronounced that the 1998 Master Settlement Agreement between the US government and tobacco companies banned the advertising of tobacco products on billboards altogether [31]. But it is vital in such assessments to separate the content advertised (i.e., the message) from the medium of advertising (i.e., the messenger). Such robust exposure potential actually can be used to benefit communities by featuring advertisements which support health and wellness, encouraging, for example, preventive care measures, routine health screening, proper diet and nutrition, regular physical fitness, and the like. Given health disparities and the reduced health status of the underprivileged, coupled with high exposures provided by billboard advertisements, opportunities emerge to deploy the medium in a manner that connects these difficult to reach audiences with information of value to personal and community health. Intensive efforts to present healthful images and practices by a range of health and wellness organizations could go a long way toward rectifying much of the previous harm resulting from the advertising of vice products via billboards in disadvantaged neighborhoods.

Contextual matters are magnified by research findings which indicate that the disadvantaged possess an inherent receptiveness toward billboard advertising and the associated content provided by the medium. Specifically, the impact and influence of billboard advertising has been found to be greatest among those possessing reduced levels of education and lower levels of income, two of the most notable characteristics which distinguish the disadvantaged from more privileged individuals. Further, the impact and influence of the medium was found to generally be more pronounced among members of the black population and less pronounced among their white counterparts, an important finding, given wide education and income disparities between the two groups, with blacks being far more likely than whites to have reduced levels of both. As education and income levels increase, the power of billboards has been found to diminish considerably, something noted to occur with other advertising media and often attributed to increased skepticism, broader world views, greater access to information sources, and related characteristics associated with better educated, higher earning individuals [16].

These research findings are very intriguing in that they suggest that the disadvantaged possess elevated perspectives of the medium and its informational content, relying on it more so than their more privileged counterparts. This goes beyond contextual matters where geographic proximity results in high exposure which affords the potential for advertising impact. Instead, this suggests that, for disadvantaged populations, advertising impact is tied to an openness or responsiveness associated with the medium. When matters of context are paired with matters of receptiveness, it becomes very apparent that billboard advertising offers a significant inroad into the lives of the disadvantaged, increasing the likelihood of successfully reaching intended audiences by deploying this particular medium of communication. Given hardships faced by health and medical institutions to communicate with impoverished populations, this is a point that certainly should not be lost when formulating plans to engage the less fortunate.

## Conclusions

Given the limited means of communication available to the disadvantaged, coupled with desires of health and medical organizations to reach out to these audiences to communicate helpful information, knowledge of a medium of communication that diminishes associated barriers and hardships is most welcomed. Of course, billboard advertising certainly is not a perfect solution for connecting with all underserved populations. It, for example, would not be very effective for reaching rural audiences, as billboard density is very low in less populated areas due to diminished traffic counts and fewer advertising exposure opportunities. But for the disadvantaged residing in urban areas, and particularly densely-populated, inner-city neighborhoods, Willis-Knighton Health System’s experiences and insights, coupled with findings from the literature, suggest that billboards offer productive pathways for reaching out to these challenged populations.

Perhaps most encouraging is the prospect, courtesy of the insights supplied in this article, that an increasing number of institutions dedicated to health and wellness would decide to deploy billboard advertising as a means of reaching the underserved. In many disadvantaged communities, positive communications are few and far between, and billboard advertisements which heavily market goods and services that exacerbate diminished health outcomes, promote high-risk behaviors, and the like certainly do not add value in these troubled settings. Messages of health, wellness, and associated opportunities would offer a refreshing change that could possibly lead to tangible improvements in personal and community health. Billboard advertising appears to be well suited to make a positive difference when deployed by institutions seeking to elevate the health status of the disadvantaged.
